# The Growing Complexity of Cancer Cell Response to DNA-Damaging Agents: Caspase 3 Mediates Cell Death or Survival?

**DOI:** 10.3390/ijms17050708

**Published:** 2016-05-11

**Authors:** Razmik Mirzayans, Bonnie Andrais, Piyush Kumar, David Murray

**Affiliations:** Department of Oncology, University of Alberta, Cross Cancer Institute, Edmonton, AB T6G 1Z2, Canada; bonnie.andrais@ahs.ca (B.A.); pkumar@ualberta.ca (P.K.); david.murray5@ahs.ca (D.M.)

**Keywords:** Caspase 3, p21^WAF1^ (CDKN1A), prostaglandin E_2_, DNA damage response, ionizing radiation, DNA double-strand breaks, γH2AX foci, apoptosis, premature senescence, multinucleation

## Abstract

It is widely stated that wild-type p53 either mediates the activation of cell cycle checkpoints to facilitate DNA repair and promote cell survival, or orchestrates apoptotic cell death following exposure to cancer therapeutic agents. This reigning paradigm has been challenged by numerous discoveries with different human cell types, including solid tumor-derived cell lines. Thus, activation of the p53 signaling pathway by ionizing radiation and other DNA-damaging agents hinders apoptosis and triggers growth arrest (e.g., through premature senescence) in some genetic backgrounds; such growth arrested cells remain viable, secrete growth-promoting factors, and give rise to progeny with stem cell-like properties. In addition, caspase 3, which is best known for its role in the execution phase of apoptosis, has been recently reported to facilitate (rather than suppress) DNA damage-induced genomic instability and carcinogenesis. This observation is consistent with an earlier report demonstrating that caspase 3 mediates secretion of the pro-survival factor prostaglandin E_2_, which in turn promotes enrichment of tumor repopulating cells. In this article, we review these and related discoveries and point out novel cancer therapeutic strategies. One of our objectives is to demonstrate the growing complexity of the DNA damage response beyond the conventional “repair and survive, or die” hypothesis.

## 1. Introduction

Clonogenic inactivation of cancer cells after exposure to therapeutic agents is governed by complex genome-surveillance mechanisms. Although these mechanisms are not fully understood, it is now well established that genotoxic stress activates early responses such as cell cycle checkpoints and DNA repair pathways which serve to promote cell survival by eliminating genomic injury, as well as late responses that eliminate cells that have developed genomic instability from the proliferating population [1,2]. Cancer cells exposed to DNA-damaging agents may be inactivated through apoptosis and other modes of cell death (e.g., necrosis) [3,4,5]; they may exhibit a prolonged blockage of proliferation [6,7]; or they may execute aberrant mitosis and give rise to aneuploid progeny, a subset of which may exhibit extended life span and result in cancer relapse [7,8,9,10,11]. Elucidating the individual contribution of these responses to the final eradication of clonogenic cancer cells and exploring the fate of cancer cells that “escape” death following genotoxic stress are crucial for designing effective strategies for cancer therapy.

Many different *in vitro* methods are available for identifying new drugs with potential anti-cancer properties when used alone or in combination with conventional therapeutic agents. The colony formation assay, developed sixty years ago [12,13,14], has since been used as the “gold standard” for evaluating radiosensitivity and chemosensitivity. More recently, numerous colorimetric 96-well plate assays (e.g., MTT and MTS) have been developed that have facilitated high-throughput screening of drugs with anti-cancer properties [15,16]. Despite their ease of use, such short-term assays lack specificity; they measure the sum of transient cell cycle checkpoints (pro-survival), growth arrest that may or may not be reversible, and loss of viability (death). Unfortunately, the results obtained with colony formation and 96-well plate assays have often been misinterpreted to reflect loss of viability and hence lethality. Furthermore, several laboratories have relied on biochemical/molecular approaches (e.g., activation of caspases, induction of pro-apoptotic genes), and sometimes even cell-free tests, as a measure of cell death.

In view of the growing complexity of signaling pathways that impact cell fate decision upon exposure to genotoxic agents, with different stress-associated proteins (e.g., caspases) mediating different and often opposing responses, the Nomenclature Committee on Cell Death (NCCD) has cautioned the authors, reviewers and editors of scientific periodicals about several caveats regarding the misuse of terminologies and concepts in the area of cell death research [17,18]. In 2009 [17], the NCCD proposed that “*a cell should be considered dead when any one of the following molecular or morphological criteria is met: (1) the cell has lost the integrity of its plasma membrane, as defined by the incorporation of vital dyes (e.g., PI) in vitro; (2) the cell, including its nucleus, has undergone complete fragmentation into discrete bodies (which are frequently referred to as ‘apoptotic bodies’); and/or (3) its corpse (or its fragments) has been engulfed by an adjacent cell in vivo. Thus, bona fide ‘dead cells’ would be different from ‘dying cells’ that have not yet concluded their demise (which can occur through a variety of biochemically distinct pathways). In particular, cells that are arrested in the cell cycle (as it occurs during senescence) should be considered as alive, and the expression ‘replicative cell death’ (which alludes to the loss of clonogenic potential), as it is frequently used by radiobiologists, should be abandoned.*”

Unfortunately, these recommendations are largely overlooked, with numerous recent reports drawing conclusions on apoptosis based only on molecular readouts (e.g., immunoblot measurement of active caspase 3) without actually determining cell demise. It is therefore not surprising that conflicting results continue to be reported. For solid tumor-derived cell lines exposed to ionizing radiation, for example, computational studies that are largely based on the two-arm model of DNA damage response (repair and survive or die) have predicted the significance of p53 in promoting apoptosis [19,20,21,22,23], whereas experimental studies have demonstrated the significance of p53 signaling in down-regulating apoptosis [24,25,26,27,28,29,30,31] and promoting a senescence-like growth arrest [25,32,33,34,35,36,37], which is now known to be reversible [7,10,11,38,39].

Given the well-characterized role of caspase 3 as an apoptosis executioner in both intrinsic and extrinsic pathways, many laboratories, ours included [40], have merely focused on its pro-apoptotic property. However, a search of the literature for other functions of caspase 3 reveals another picture. An overwhelming number of reports demonstrate non-apoptotic roles for caspase 3 in different biological systems. For example, as extensively discussed by D’Amelio *et al.* [41,42], caspase 3 plays an important role in physiological processes such as neurodevelopment and differentiation that do not cause cell death. Apoptosis-independent function of caspase 3 has also been implicated in Alzheimer’s, Parkinson’s and other neurodegenerative diseases [41,42,43]. In addition, caspase 3 has been recently demonstrated to stimulate the repopulation of tumors undergoing cancer therapy [44,45] and to promote genomic instability and tumorigenesis [46].

**Figure 1:**
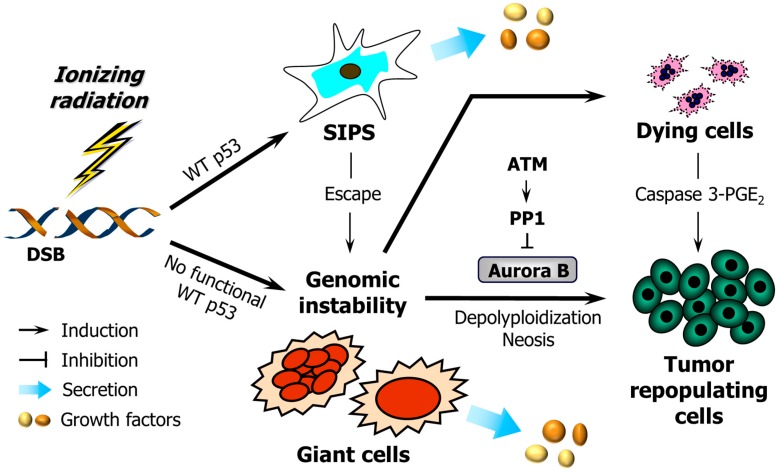
The DNA damage response of human cells with differing p53 status discussed in this article. Ionizing radiation triggers growth arrest through stress-induced premature senescence (SIPS) in p53 wild-type (WT) cells, and the development of giant cells (containing multiple nuclei or a single enlarged nucleus) within cultures of cancer cells lacking wild-type p53 function. In addition, a proportion of p53 WT cells “escapes” from SIPS and gives rise to giant cells. While some giant cells may die through apoptosis, others may undergo complex genome-reduction processes (e.g., depolyploidization and neosis), ultimately giving rise to rapidly-proliferating progeny. The mitotic kinase Aurora B plays an important role in regulating the survival of giant cells. ATM may prevent the propagation of giant cells and their descendants by activating protein phosphatase 1 (PP1) and inhibiting Aurora B kinase activity [37,47]. Caspase 3 either functions as the executioner caspase in the apoptotic pathway or, paradoxically, promotes cell survival by mediating prostaglandin E_2_ (PGE_2_) secretion. DSB, double-strand break; ATM, ataxia telangiectasia mutated.

Herein, we review the current state of understanding regarding the long-term fate of cancer cells upon exposure to DNA-damaging agents and consider recent papers by Huang *et al.* [44] and Liu *et al.* [46] demonstrating pro-survival functions of caspase 3. Our objective is to briefly review the compelling experimental data that support the complex stress-induced responses illustrated in Figure 1.

## 2. Cancer Cell Response to Genotoxic Stress: Reversible Growth Arrest or Cell Death?

### 2.1. Stress-Induced Growth Arrest in p53 Wild-Type Cells

The p53 protein, also known colloquially as the “guardian of genome” [48], serves to eliminate DNA damage from cells following genotoxic stress by accelerating DNA repair processes and activating transient cell cycle checkpoints to facilitate repair. When the damage is severe, p53 can trigger apoptotic cell death either directly through its polyproline region [49], or indirectly through transcriptionally upregulating pro-apoptotic proteins such as the BH3-only family (PUMA, NOXA and BAX), and downregulating anti-apoptotic proteins such as BCL-2 and survivin [50,51,52]. Somewhat paradoxically, p53 also transcriptionally activates p21^WAF1^ (hereafter, p21), a multifunctional tumor suppressor that, among other activities, can down-regulate apoptosis and orchestrate growth arrest through stress-induced premature senescence (SIPS) [1]. SIPS is characterized by flattened and enlarged cell morphology in cells that retain viability but cease to divide for extended periods after genotoxic stress. In addition, p53 can suppress apoptosis through p21-independent mechanisms, such as promoting DNA repair and activating the pRB pathway [24]. Current evidence indicates that the biological outputs orchestrated by p53 are context dependent and may be influenced by numerous factors, including the cell type and the nature of the genotoxic insult. In non-cancerous human dermal fibroblast strains and solid tumor-derived cell lines, activation of the p53–p21 pathway confers apoptosis resistance and promotes SIPS upon exposure to ionizing radiation [1].

Although sustained growth arrest is one of the hallmarks of SIPS, this response is ultimately reversible, at least in malignant cell types. Accordingly, SIPS appears to represent a mechanism by which tumor cells evade cell death consequent to radiotherapy and chemotherapy, allowing for their re-emergence and disease recurrence. The reversibility of SIPS and other modes of senescence has been the subject of a recent review article by Chakradeo *et al.* [7].

### 2.2. Apoptotic Threshold in p53 Wild-Type Human Cells Exposed to Genotoxic Agents

Numerous studies reported since the 1990s, some from our laboratory [53,54,55,56,57], have established the presence of a threshold mechanism for stress-induced growth arrest *versus* apoptosis in human cells with different p53 status, with moderate doses of DNA-damaging agents predominantly (if not solely) triggering growth arrest (e.g., SIPS) in some cell types. This threshold effect has been observed for ionizing radiation [54,55,56,57,58,59], 254 nm ultraviolet light (UV) [53,54,60,61] and chemotherapeutic agents [62,63,64,65,66,67,68,69].

**Figure 2:**
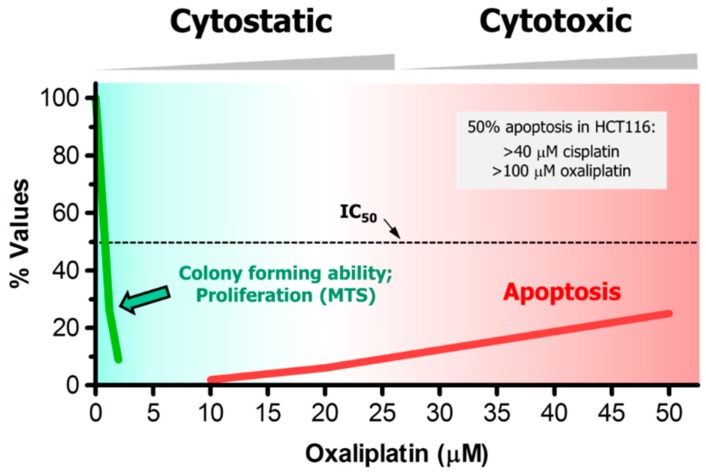
Dose-dependent responses induced by oxaliplatin in HCT116 cells, measured by colony formation [68], cell proliferation (e.g., MTS) and apoptosis assays [67]. Such large discrepancy between growth-inhibitory and apoptosis-inducing concentrations has also been reported for this and other solid tumor-derived cell lines after treatment with cisplatin. In HCT116 cells, for example, <5 μM [68] and >40 μM [66] cisplatin concentrations induced 50% effect (IC_50_) in colony formation and apoptosis assays, respectively.

In HCT116 colon carcinoma cells treated with oxaliplatin, for example, moderate concentrations that are typically used in the colony formation assay (e.g., <5 μM) [68] result in significant growth arrest (e.g., determined by the MTS cell proliferation assay) but virtually no apoptosis [67], whereas >20 μM concentrations of oxaliplatin are required to trigger apoptosis [67] (also see Figure 2). Similar discrepancy between growth-inhibitory and apoptosis-inducing concentrations has been reported for cisplatin (reviewed in [66] for a large number of cancer cell lines), paclitaxel [65] and doxorubicin [62,63,64]. When examined, moderate (sub-apoptotic) concentrations of these agents trigger a high degree of SIPS [66,70,71].

**Figure 3:**
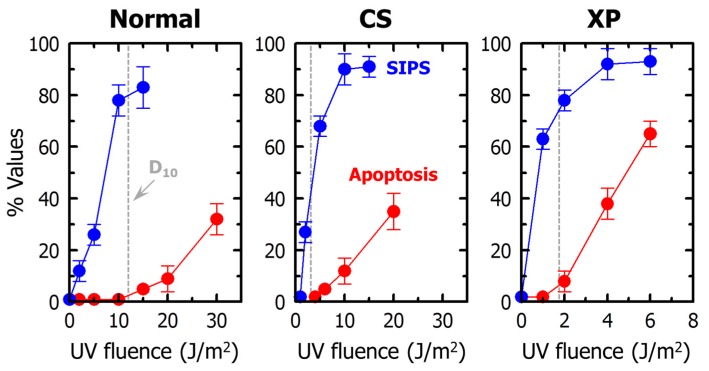
Induction of SIPS (blue circles) and apoptosis (red circles) by UV in human normal, Cockayne syndrome (CS), and xeroderma pigmentosum (XP) fibroblast strains. Dashed lines show fluences of UV that induced 90% effect in the colony formation assay. The results are reproduced from [54] with permission. D_10_, 10% “survival” in the clonogenic assay.

For UV, the presence of an apoptotic threshold is observed not only in nucleotide excision repair-proficient (normal) cells, but also in repair-deficient cells derived from patients with the neurodegenerative disorders xeroderma pigmentosum and Cockayne syndrome [54,63] (also see Figure 3). In both excision repair-proficient and -deficient cells, loss of colony forming ability following UV exposure is largely (if not totally) associated with SIPS.

For ionizing radiation, some p53 wild-type cells exhibit only marginal (if any) apoptotic cell death even after exposure to extremely high doses. For example, ataxia telangiectasia (ATM-deficient) human skin fibroblasts [55] and A172 human malignant glioma cells [57] are remarkably resistant to undergoing apoptosis upon exposure to radiation doses as high as 50 Gy and 280 Gy, respectively.

Some reports do suggest the induction of apoptosis after treatment with moderate doses of some genotoxic agents. Most of these reports, however, did not follow the NCCD recommendations to distinguish between dying cells (e.g., exhibiting active caspase 3, which may promote apoptosis or survival depending on context) and dead cells.

In addition to these responses, genotoxic stress has been reported to induce multinucleation in some cell types (not included in Figure 2 and Figure 3). For example, multinucleated giant cells can emerge from cancer cell cultures undergoing SIPS after exposure to genotoxic agents [1] (also see Figure 1). In addition, Kaur *et al.* [9] have recently demonstrated that radiation exposure triggers homotypic cell fusions of innately resistant human glioma cells and that this response is associated with their survival and recurrence. It is important to note that some authors consider multinucleation to reflect death through apoptosis or mitotic catastrophe, which is not consistent with the experimental data (see Section 3.1).

### 2.3. Stress-Induced Growth Arrest in p53-Deficient Cells

The observation that the *TP53* gene is mutated in ~50% of human cancers led to a great deal of research toward elucidating the impact of p53 status on the degree of cell killing induced by cancer therapeutic agents in different human cell types [72]. This knowledge is essential in part because of the possibility of transiently targeting p53 (e.g., by the use of small molecule modulators of p53) for selectively augmenting the sensitivity of tumor cells to such agents and hence improving the outcome of cancer therapy. As recently reviewed by us [37] as well as others [73,74], this approach has met with limited success despite extensive efforts over the past three decades. It has become increasingly evident that the p53 status of solid tumor-derived cancer cells only marginally, if at all, influences the degree of apoptosis or other modes of cell death following exposure to moderate, clinically-relevant doses of therapeutic agents. Halacli *et al.* [26], for example, demonstrated that the HCT116 cell line and its p53 knockout derivative (hereafter, HCT116p53−/−) exhibit comparable levels of apoptosis in response to ionizing radiation, and concluded that the radiation-induced apoptosis is p53-independent in this widely used colon carcinoma model. It is important to note that radiation exposure of HCT116p53−/− cells results in robust activation of caspase 3 [25] but does not confer radiosensitivity in the colony formation assay [75] when compared to parental HCT116 cells.

Thus, if not apoptosis, then what is the long-term fate of p53-deficient cells consequent to radiation exposure? Instead of promoting cell death in response to stress, loss of wild-type p53 function can result in a switch from p53/p21-dependent growth arrest (SIPS) to p16-directed SIPS (reviewed in [76]). Unfortunately, as mentioned above, a proportion of solid cancer-derived cells undergoing SIPS acquire the ability to escape from growth arrest and give rise to therapy-resistant progeny [71,77,78]. Furthermore, studies with ionizing radiation and chemotherapeutic agents have revealed that loss of p53 is permissive for the development of endopolyploid “giant” cells (e.g., containing multiple nuclei) [8,79,80]. Endopolyploid cancer cells can segregate their genome and return to the mitotic cycle [8,79,80]. The genome reduction process is mediated by key regulators of mitosis (e.g., aurora B kinase), meiosis (e.g., MOS), and self-renewal (e.g., OCT4). In addition, it has long been recognized that endopolyploid giant cells that are formed post-treatment can undergo “neotic” cell division, which resembles the division of budding yeast, ultimately giving rise to progeny with stem cell-like properties (reviewed in [1]). This so-called “endopolyploidy-stemness” model of cancer-cell survival consequent to therapeutic exposure has been documented with different types of human cancer cell lines (reviewed in [1,80]) as well as with short-term (2–3 weeks) cultures of primary human breast cancer specimens [81].

## 3. Growth-Arrested Cells Secrete Growth-Promoting Factors

### 3.1. Factors Secreted by Multinucleated Giant Cells

Sixty years ago, a series of landmark studies reported by Puck and Marcus [12] demonstrated that ionizing radiation triggers the development of multinucleated giant cells that remain viable and secrete cell-growth promoting factors. The authors used the human HeLa cervical carcinoma cell line [12,13,14], which was subsequently shown to be p53 function deficient [82]. In response to high doses of ionizing radiation, virtually all cells within HeLa cell cultures were reported to become multinucleated. These observations prompted the development of the feeder layer colony formation assay, in which heavily-irradiated feeder (giant) cells serve to promote the growth of test cells. This feeder layer technique proved to be instrumental in evaluating radiosensitivity and chemosensitivity in cells with low cloning efficiency, including non-cancerous dermal fibroblast strains derived from patients with various hereditary degenerative disorders.

Puck and Marcus reported detailed evaluation of the fate of multinucleated HeLa cells upon radiation exposure [12,13,14]. These cells either ceased to divide or divided slowly in the time span of the colony forming assay, but not often enough to give rise to an aggregate of at least 50 cells, which has since been considered the cut off cell number for a “surviving” colony. In addition, viral infection of giant cells resulted in their demise.

These intriguing discoveries went largely unnoticed, and with time some authors considered multinucleation to reflect cell death. As alluded to earlier, in the past decade, several laboratories using different biological systems, both *in vitro* and *in vivo*, provided evidence that multinucleated giant cancer cells that are created after genotoxic stress not only remain viable, but also serve as a “factory” for the genesis of progeny that exhibit stem cell-like properties. Furthermore, it is becoming increasingly evident that the creation of multinucleated giant cells consequent to genotoxic stress is not an infrequent response, at least for solid tumors [9], and that multinucleated cancer cells can contribute to disease relapse [9,10,11,39].

### 3.2. Factors Secreted by Cells Undergoing SIPS

In addition to a sustained growth-arrested response, cells undergoing SIPS acquire the ability to secrete factors that can promote malignant features such as proliferation and invasiveness in cell culture models and tumor development *in vivo*. This phenotype has been termed the “senescence-associated secretory phenotype” (SASP) and is now considered to represent the darkest side of tumor suppression, even for non-cancerous skin fibroblasts [83]. The SASP includes several families of soluble and insoluble factors that can affect surrounding cells by activating various cell surface receptors and corresponding signal transduction pathways that may lead to cancer and other pathologies. SASP factors can broadly be divided into three categories: soluble signaling factors (e.g., interleukins), secreted proteases (e.g., matrix metalloproteinases), and secreted molecules other than proteins (e.g., reactive oxygen species). The types and functions of different SASP factors have been extensively reviewed [83,84] and will not be considered further.

## 4. Pro-Survival Function of Caspase 3: Mediating Prostaglandin E_2_ Secretion, Genomic Instability and Carcinogenesis

Caspase 3 and other executioner caspases have long been recognized as the key proteases mediating cell demolition during apoptotic cell death [85,86]. Caspases have also been extensively studied for their ability to modulate signal transduction inside cells, by positively or negatively regulating kinases, phosphatases and other signaling molecules. Interestingly, a series of recent studies have highlighted the involvement of executioner caspases in triggering a “bystander” response, stimulating proliferation of neighboring cells [44,45,46,87,88,89,90]. We will focus on two articles on caspase 3, reported by Huang *et al.* [44] and Liu *et al.* [46]. The former article shows that activation of caspase 3 in a small number of cells after exposure to ionizing radiation results in growth of neighboring cells not exposed to radiation, and that this response involves caspase 3-mediated secretion of PGE_2_. The latter article demonstrates that active caspase 3 promotes genomic instability in cultured cells and tumor growth in animals.

### 4.1. Caspase 3 Mediates Secretion of Pro-Survival Factors

The large numbers of cells within a tumor that are killed during cytotoxic therapy are engulfed by macrophages and other scavenger cells. The surviving tumor cells, however, are presumed to gradually proliferate and ultimately re-establish the tumor. In addition, it has long been recognized that tumors respond to therapy by initiating a process by which the few surviving cells are triggered to proliferate at a markedly accelerated pace and rapidly repopulate the tumor. Huang *et al.* [44] explored the molecular basis for this accelerated repopulation process.

These authors first examined whether heavily irradiated (e.g., 10 Gy) tumor cells can stimulate rapid tumor repopulation in their *in vitro* and *in vivo* model systems. They used firefly luciferase (Fluc)-labelled 4T1 mouse breast cancer cells and monitored growth of these cells through non-invasive bioluminescence imaging. As expected from previous studies with other cell types (see above), 4T1-Fluc cells grew significantly faster when seeded onto irradiated feeder cells than when seeded alone. Similarly, in animals injected with a mix of unirradiated Fluc-labeled cells and “feeder” (unlabeled but heavily irradiated) or control (unlabeled and unirradiated) tumor cells, the presence of irradiated feeder cells markedly increased the growth of Fluc-labeled tumor cells.

Given that severe genotoxic stress triggers activation of caspases and that caspase 3 exerts both pro-apoptotic and pro-survival effects depending on context, Huang *et al.* [44] determined whether this caspase is responsible for mediating tumor repopulation. This was accomplished by the use of isogenic cell lines with differing caspase 3 status, generated by either expressing exogenous caspase 3 in caspase 3-deficient cells (e.g., MCF7 breast carcinoma) or shRNA-mediated knockdown of caspase 3 in caspase 3-proficient cells (e.g., 4T1). Compared to caspase 3-deficient cells, isogenic caspase 3-proficient cells were remarkably more effective in promoting growth of irradiated cancer cells *in vitro* and growth of tumors *in vivo*.

Next Huang *et al.* [44] determined whether PGE_2_ is implicated in the tumor repopulation process. The rationale for this hypothesis was that PGE_2_ is known to stimulate tumor growth and stem cell proliferation. Moreover, PGE_2_ functions downstream in a caspase 3-activated signaling pathway involving cytosolic calcium-independent phospholipase A_2_ (iPLA_2_) and arachidonic acid. In a series of experiments involving isogenic cell lines with different expression levels of caspase 3 and iPLA_2_, it was shown that the tumor repopulation process that is mediated by caspase 3 following radiation exposure is mediated by the iPLA_2_-arachidonic acid-PGE_2_ axis.

Huang *et al.* [44] also determined the relevance of these preclinical observations to tumor repopulation in human cancer treatment by examining caspase 3 status in two cohorts of human subjects with cancer: 48 patients with advanced stage breast cancer and 39 patients with head and neck cancer treated with radiotherapy or chemo-radiotherapy. Levels of activated caspase 3 in tumor tissue were found to predict worse treatment outcome.

Collectively, these observations demonstrate the existence of a stress-triggered tumor repopulation pathway in which caspase 3 and PGE_2_ play major roles.

### 4.2. Caspase 3 Activation Can Promote Genomic Instability and Carcinogenesis

In the studies reported by Huang *et al.* [44], which involved cell exposure to high doses of ionizing radiation, it was uncertain whether the caspase 3-mediated responses were associated with apoptosis, other modes of cell death, and/or growth arrest (e.g., reflecting SIPS). Liu *et al.* [46], on the other hand, used a sub-lethal dose of ionizing radiation (0.5 Gy) to test their hypothesis that caspase 3 might facilitate carcinogenesis by inducing genomic instability. Sublethal activation of caspase 3 in cultured cells was shown to promote persistent DNA double-strand breaks (DSBs), a high frequency of chromosome aberrations, and acquisition of the ability to grow in an anchorage-independent manner in soft agar. In addition, skin carcinogenesis induced following treatment with 7,12-dimethylbenz(a)anthracene (DMBA) plus 12-*O*-tetradecanoyphorbol-13-acetate was significantly reduced in mice that were genetically deficient in caspase 3. These observations revealed a surprising and unconventional role for caspase 3 in the DNA damage response; namely, its ability to cause and sustain genomic instability and facilitate oncogenic transformation in response to DNA damage.

## 5. Is Caspase 3-Mediated Secretion of Pro-Survival Factors Associated with Dying Cells, Dead Cells, or Both?

Liu *et al.* [46] determined the impact of caspase 3 on the fate of MCF10A human mammary cells after exposure to a moderate dose (0.5 Gy) of high-energy particle (^56^Fe ion) irradiation. Employing an elegantly constructed caspase 3 reporter system and fluorescence-activated cell sorting, immunoblotting and immunostaining techniques, these authors showed that radiation exposure triggers significant caspase-3 activation in a persistent manner (e.g., up to two weeks post-irradiation) as well as cytochrome C release, which is often associated with apoptosis. Despite these effects, the cells exhibited normal morphology with no signs of apoptosis post-irradiation. As concluded by the authors, these data contradict the popular view that damaged cells with active caspase 3 are destined to die.

**Figure 4:**
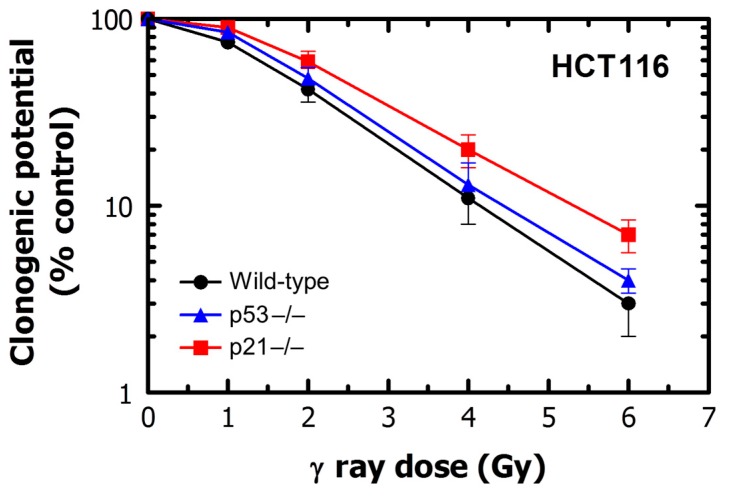
Radiosensitivity of HCT116 wild-type, HCT116p53−/− and HCT116p21−/− cultures evaluated by colony formation assay.

These observations are reminiscent of the results reported for the HCT116 colon carcinoma cell line and its p53 knockout (HCT116p53−/−) and p21 knockout (HCT116p21−/−) derivatives. As mentioned earlier, loss of p53 in HCT116 cells results in robust activation of caspase 3 following exposure to ionizing radiation (^137^Cs γ rays) [54] but does not lead to increased radiosensitivity in the colony formation assay [75]. Interestingly, activation of caspase 3 in HCT116p53−/− cells, following radiation exposure, was reported to be accompanied by poly(ADP-ribose) polymerase (PARP) cleavage, outer mitochondrial membrane depolarization and cytochrome C release, all of which are often used as markers of apoptosis; strikingly, however, HCT116p53−/− and parental cultures showed comparable radiosensitivity. Similar results have been reported for HCT116p21−/− cells. Thus, HCT116p21−/− cells respond to ionizing radiation by exhibiting caspase 3 activation and PARP cleavage together with mitochondrial and cytoplasmic association [54], but in the colony formation assay they exhibit a small degree of *radioresistance* (and not increased radiosensitivity, as would be expected for apoptosis-sensitive cells) when compared to wild-type cells [91]. We have confirmed the radiosensitivity responses of these three cell lines using the colony formation assay (Figure 4). Based on these observations, it is reasonable to conclude that activation of caspase 3 under these conditions is not associated with cell death, probably because caspase 3 does not “push” the cells beyond the point-of-no-return.

This conclusion is consistent with the NCCD recommendations published in 2009 [17] (also see Section 1 above), and updated recommendations published in 2012 [18], stating that “… a *cell death-associated biochemical process can develop at a sublethal or transient level, which does not lead to the cell demise. Thus, while full-blown mitochondrial outer membrane permeabilization (MOMP) constitutes a point-of-no-return of intrinsic apoptosis, limited extents of MOMP (*i.e.*, concerning a fraction of the mitochondrial pool) and the consequent (localized) activation of caspase-3 have been shown to participate in several cell death-unrelated programs.*”

Studies reported by Huang *et al.* [44] were mainly performed with the 4T1 mouse breast cancer cell line; some experiments were also performed with mouse embryonic fibroblasts (MEFs) and human MCF7 breast carcinoma cells. The radiosensitivity of these cells was determined in culture and after implantation into live animals. For both *in vitro* and *in vivo* experiments reported in the main text, radiosensitivity was evaluated by counting the cells at various times after exposure to relatively high doses of ionizing radiation (between 8 and 12 Gy). The authors considered the growth inhibition seen under these conditions to reflect lethality, which has led to the notion that dead (apoptotic) cells can secrete growth-promoting factors [44,45]. However, as mentioned earlier, studies with human cells with differing genetic backgrounds have established that such radiation doses typically result in a high degree of growth arrest (through SIPS and/or multinucleation), but marginal (if any) cell demise. Unfortunately, Huang *et al.* [44] did not determine the fate of these irradiated cells in terms of extended growth arrest (e.g., SIPS) *versus* cell death.

In the supplementary material, Huang *et al.* [44] reported radiosensitivity studies with isogenic caspase 3-proficient and -deficient cell lines. Cultures with differing caspase 3 status showed identical radiosensitivity in the colony formation assay (their supplementary Figure 5), which does not appear to be consistent with caspase 3 promoting cell death under these conditions. Furthermore, fluorescence (DAPI) images corresponding to these cultures that were exposed to a high dose (10 Gy) of ionizing radiation did not appear to show the presence of cells with apoptotic morphology (condensed/fragmented nuclei) (their supplementary Figures S6–S8). Huang *et al.* [44] also performed the TUNEL assay post-irradiation (10 Gy) and observed TUNEL-positivity in only a small proportion of cells (e.g., ~6% of MCF7casp3 cells and ~13% of 4T1 cells) within caspase 3-expressing cultures and absence of TUNEL-positive cells within caspase 3-deficient cultures. Although apoptotic cells are positive in the TUNEL assay, it is important to note that this assay detects DSBs that might or might not be associated with apoptosis. Furuta *et al.* [92], for example, demonstrated that TUNEL staining after treatment with UCN-01 and camptothecin, used singly or in combination, is induced by DSBs unrelated to apoptosis. It is also important to note that induction of caspase 3 has been recently shown to be accompanied by persistent DNA DSBs in the absence of apoptosis [44].

In short, although there is compelling experimental data demonstrating a growth stimulating role for caspase 3 in the DNA damage response through mediating secretion of PGE_2_ and other pro-survival factors, whether apoptotic (dead) cells that have crossed the point-of-no-return are also capable of releasing such factors remains to be elucidated. In addition, it is currently unknown whether executioner caspases participate in the SASP program associated with cells undergoing SIPS, and whether such caspases are involved, at least in part, in mediating secretion of growth-stimulating factors by multinucleated giant cells.

## 6. Caspase 3 and p21 Interaction in the DNA Damage Response

**Figure 5:**
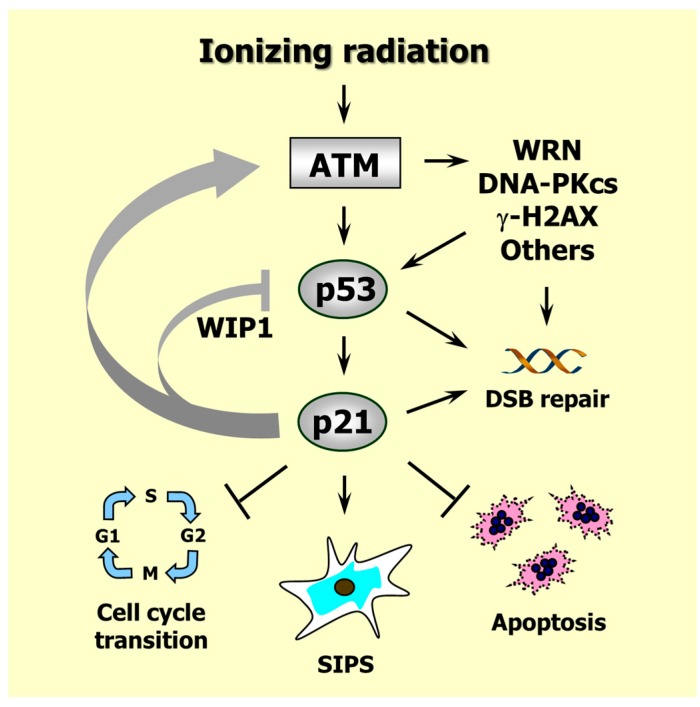
Responses induced by ionizing radiation in p53 wild-type human solid tumor-derived cell lines. Arrows indicate stimulation and T-shaped lines indicate inhibition. Radiation exposure results in ATM-dependent activation of several proteins (e.g., p53, WRN, DNA-PKcs) that play important roles in DSB repair, as well as p53-mediated activation of p21 that suppresses apoptosis and activates cell cycle checkpoints. Proper activation of these events provides time for the repair of potentially cytotoxic and mutagenic lesions. Persistence of DNA damage leads to sustained induction of p21 which downregulates p53 (e.g., through WIP1), suppresses apoptosis and triggers SIPS. Positive feedback loops between ATM and p21 ensure the maintenance of the SIPS response for extended times (e.g., several months in culture). For further details, consult [31,37]. WRN, Werner’s syndrome protein; DNA-PKcs, DNA-dependent protein kinase catalytic subunit; γ-H2AX, H2A variant histone H2AX phosphorylated on Ser 139; WIP1, wild-type p53-induced phosphatase 1.

The p21 protein is the founding member of the CIP/KIP family of cyclin-dependent kinase (CDK) inhibitors and plays a major role in the DNA damage response [1,93] (also see Figure 5). Following genotoxic stress, p21 is transcriptionally upregulated by p53 and activates cell cycle checkpoints, promotes DNA repair, downregulates apoptosis, represses mitotic genes, upregulates senescence genes, and triggers growth arrest through SIPS depending on context. This multifunctional protein also forms a positive regulatory loop with ATM, an important kinase that functions upstream in the radiation responsive DNA damage surveillance network. The ATM-p21 positive feedback loop is essential for the maintenance of growth arrest associated with SIPS [94]. In addition, studies with animal models have revealed that cooperation of ATM and p21 is essential to suppress aneuploidy and subsequent tumor development [95].

Although ATM is one of the main kinases that mediate stabilization and activation of p53, p21 is surprisingly found to downregulate p53. This effect is mediated by various mechanisms, including p21-dependent upregulation of the phosphatase “wild-type p53-induced phosphatase 1” (WIP1), a p53 inhibitor that plays a key role in controlling p53 dynamics after exposure to ionizing radiation [31].

In addition, p21 also interacts with caspase 3 in the DNA damage response, resulting in repression of apoptosis [96,97]. Whether this property of p21 is associated with caspase 3-mediated secretion of pro-survival factors discussed above remains an open question. While intact p21 inhibits apoptosis, a 15-kDa cleavage product of p21 has been paradoxically reported to exert pro-apoptotic function. As discussed previously [37], caspase 3 mediates p21 cleavage and generates this 15 kDa fragment which facilitates caspase 3-directed apoptosis.

Thus, one mechanism by which caspase 3 exerts its influence on cell fate upon genotoxic stress is through interacting with p21, a master regulator of the DNA damage response.

## 7. Conclusions and Perspectives

Treatment of solid tumor-derived cells with moderate, clinically relevant doses of anti-cancer agents triggers a growth-arrested response that is predominantly associated with SIPS and multinucleation in p53-proficient and -deficient cells, respectively. Such growth-arrested cells remain viable and can contribute to cancer progression and recurrence through secreting growth-promoting factors, as well as by giving rise to tumor repopulating progeny. Unfortunately, similar to growth arrested cells, “dying” cancer cells can also contribute to cancer relapse by activating the caspase 3-PGE_2_ survival pathway. These observations demonstrate the growing complexity of cancer cell response to genotoxic stress beyond the conventional “repair and survive, or die” hypothesis. They also underscore the NCCD recommendations in terms of distinguishing between dying cells (that may not cross the point-of-no-return) *versus* dead cells.

Employing a sub-lethal dose of ionizing radiation, Liu *et al.* [46] demonstrated that in the absence of apoptosis, radiation exposure results in activation of caspase 3 and subsequent release of factors that trigger genomic instability and tumor formation. Huang *et al.* [44], on the other hand, used high doses of ionizing radiation and concluded that caspase 3-mediated secretion of PGE_2_ and tumor repopulation is associated with apoptotic cells. Considering the NCCD recommendations, we suggest that caution should be exercised in the interpretation of these results because the authors did not distinguish between dying and dead cells. Importantly, in caspase 3-reconstituted MCF7 cultures, a 10-Gy dose of radiation caused ~0.1% survival (*i.e.*, growth arrest plus death in ~99.9% of cells) in the colony formation assay, but resulted in TUNEL positivity in only a small fraction of the cells (e.g., ~6% “kill”). Thus, it is uncertain whether the caspase 3-PGE_2_ response is triggered by a small proportion (~6%) of TUNEL positive (presumably apoptotic) cells, a large proportion (~94%) of TUNEL negative (but growth arrested) cells, or both. Further studies are warranted to address this question.

The intriguing observations reviewed in this article suggest that targeting growth-arrested cancer cells might represent an effective strategy for combating this devastating disease. Consistent with this notion, Crescenzi *et al.* [94] reported that down-regulating either ATM or p21 in cancer cells that have undergone SIPS following chemotherapeutic exposure results in their death. For targeting multinucleated cancer cells, the landmark study reported by Puck and Marcus sixty years ago [12] demonstrated that viral infection promotes death of multinucleated cervical carcinoma (HeLa) cells. Recently, Shah *et al.* [98] reported that downregulating the Bcl-XL/Bcl-2 pathway in multinucleated colon carcinoma cells (HCT116) results in their demise. The authors have provided a movie (supplementary material in [98]) demonstrating rapid death of multinucleated giant cells through the application of ABT-263, an orally bioavailable small-molecule inhibitor of Bcl-X, Bcl-2 and Bcl-w.

However, in order for targeting growth-arrested cancer cells to have significant impact on the outcome of cancer therapy, it needs to be accompanied by approaches that block the pro-survival function of caspase 3 in general, and inhibit PGE_2_ in particular. To this end, recent work reported by Kurtova *et al.* [99] is intriguing. These authors investigated the molecular mechanisms by which tumors become progressively unresponsive after multiple treatment cycles in some patients. Using human bladder cancer xenografts, Kurtova *et al.* [99] showed that cancer stem cells can contribute to therapy resistance by triggering a proliferative response to repopulate residual tumors between chemotherapy cycles, and that this response is related to PGE_2_ release. This repopulation was abrogated by treatment with a PGE_2_-neutralizing antibody and celecoxib, a pharmacological inhibitor of cyclooxygenase-2 (the enzyme that mediates PGE_2_ production). *In vivo* administration of celecoxib effectively attenuated the progressive manifestation of chemoresistance in xenograft tumors derived from a bladder cancer patient who was resistant to chemotherapy. These findings support an adjunctive approach to enhancing the outcomes of conventional cancer therapies by targeting early tumor repopulation.
